# The FOXQ1/SSBP2 Regulatory Axis Drives Malignant Progression of Melanoma

**DOI:** 10.1002/mc.70126

**Published:** 2026-08-30

**Authors:** Xiaoyu Zhang, Yu Hou, Jiahui Liu, Yuehua Han, Jiangang Feng

**Affiliations:** ^1^ Department of Orthopedics The Fourth Hospital of Hebei Medical University Shijiazhuang China; ^2^ Department of Orthopaedic Surgery Affiliated Hospital of Hebei University Baoding China

**Keywords:** cell proliferation, FOXQ1, melanoma, migration, SSBP2

## Abstract

Melanoma is a highly aggressive skin cancer with poor prognosis, often linked to excessive UV exposure. Despite advancements in immunotherapy and targeted treatments, melanoma's invasiveness and metastatic potential remain significant challenges. This study aimed to identify novel molecular targets associated with melanoma progression. Consensus clustering analysis revealed three melanoma subtypes, with the C3 subtype exhibiting the worst prognosis. Weighted gene co‐expression network analysis (WGCNA) identified FOXQ1 as a key driver in this subtype. FOXQ1 expression was elevated in clinical melanoma tissues and cell lines, and silencing FOXQ1 in vitro reduced melanoma cell proliferation, migration, and induced apoptosis. To explore its downstream targets, FOXQ1 was found to repress the expression of SSBP2, a gene inversely correlated with FOXQ1. Functional assays demonstrated that silencing SSBP2 promoted melanoma malignancy. Rescue experiments showed that knockdown of SSBP2 reversed the tumor‐suppressive effects of FOXQ1 silencing. These results suggest that the FOXQ1/SSBP2 regulatory axis plays a critical role in melanoma progression and may serve as a potential therapeutic target to improve melanoma treatment outcomes.

## 1 Introduction

Melanoma is a highly malignant tumor originating from melanocytes and can be clinically classified into several subtypes, including cutaneous melanoma, mucosal melanoma, and ocular melanoma, based on anatomical location and biological behavior. These subtypes exhibit pronounced biological heterogeneity. As one of the most metastatic human cancers, melanoma poses substantial therapeutic challenges due to its exceptional metastatic potential. Notably, melanoma accounts for approximately 90% of skin cancer–related deaths despite representing a smaller proportion of skin malignancies [1, 2]. With changes in lifestyle and global atmospheric conditions, cumulative ultraviolet (UV) radiation exposure to the skin has increased, contributing to a steady rise in melanoma incidence worldwide [3]. In Europe, the incidence of melanoma is estimated at approximately 10–25 new cases per 100,000 individuals annually [4]; in the United States, the incidence ranges from 20 to 30 per 100,000 individuals [5]; and in Australia, where UV exposure is particularly intense, the incidence is remarkably high, reaching approximately 50–60 cases per 100,000 individuals [6]. This upward trend is expected to persist over the coming decades, posing a significant and growing threat to global public health. Although substantial advances have been made in immunotherapy and targeted therapeutic strategies for melanoma, its highly invasive and metastatic nature continues to limit clinical efficacy. Therefore, further clinical investigations and the identification of novel molecular targets are urgently needed to improve therapeutic outcomes and patient prognosis.

Forkhead box Q1 (FOXQ1) is a recently identified member of the forkhead (FOX) transcription factor family [7]. FOXQ1 is involved in a wide range of physiological processes, including cellular senescence [8], glucose metabolism [9], lactate production [10], and cardiac fibrosis [11]. As a transcription factor containing a conserved forkhead DNA‐binding domain, FOXQ1 has been demonstrated to play critical roles in the biological behavior of various malignancies. In particular, FOXQ1 is implicated in tumor invasion, apoptosis, and epithelial–mesenchymal transition (EMT) in multiple cancer types, including breast cancer, colorectal cancer, and intrahepatic cholangiocarcinoma [12, 13, 14, 15]. Although accumulating evidence indicates that FOXQ1 expression is closely associated with survival outcomes and prognosis in cutaneous melanoma [16], its precise functional role and underlying molecular mechanisms in melanoma progression remain largely unclear and warrant further investigation.

Single‐stranded DNA‐binding protein 2 (SSBP2) is a subunit of the single‐stranded DNA‐binding protein complex and is located on human chromosome 5q14.1. SSBP2 is involved in the maintenance of hematopoietic stem cell function and genomic stability and was previously identified as a candidate tumor suppressor gene in patients with myeloid leukemia [17, 18]. Through its highly conserved N‐terminal domain, SSBP2 interacts with LIM domain‐binding protein 1 (LDB1), a transcriptional adapter protein that has been implicated in the development of various malignancies, including head and neck cancer and colorectal cancer [19, 20]. At present, the functional role of SSBP2 in cancer remains controversial. Multiple studies have demonstrated that SSBP2 acts as a tumor suppressor in solid tumors and myeloid leukemia [21, 22, 23]. In contrast, other reports suggest that SSBP2 may function as an oncogene in glioblastoma and hepatocellular carcinoma [24, 25, 26]. To date, however, the role of SSBP2 in melanoma has not been reported. In this study, we aimed to identify key driver transcription factors through molecular subtyping and to systematically elucidate the functional significance and underlying mechanisms of the FOXQ1/SSBP2 regulatory axis in melanoma progression.

## 2 Materials and Methods

### 2.1 Public Data Sources and Preprocessing

Transcriptomic data and corresponding clinical information of melanoma used for bioinformatics analyses were obtained from the Skin Cutaneous Melanoma (SKCM) cohort of The Cancer Genome Atlas (TCGA) database. RNA sequencing (RNA‐seq) expression data in fragments per kilobase of transcript per million mapped reads (FPKM) format, along with clinical follow‐up information including age, sex, tumor stage, survival status, and follow‐up time, were downloaded from the UCSC Xena platform (https://xenabrowser.net). To improve comparability across samples, FPKM values were converted to transcripts per million (TPM), followed by log_2_ transformation [log_2_(TPM + 1)] for subsequent analyses. Samples with missing follow‐up information or an overall survival (OS) time of < 30 days were excluded to minimize potential bias from non–tumor‐related mortality. For external validation, Gene Expression Omnibus (GEO) data sets (GSE100797, GSE53118, and GSE54467) were downloaded from the National Center for Biotechnology Information (NCBI) GEO database (https://www.ncbi.nlm.nih.gov/geo/). Raw expression matrices were normalized according to the corresponding platform annotation files, and multiple probes mapping to the same gene symbol were averaged to obtain a single expression value per gene.

### 2.2 Consensus Clustering Analysis for Molecular Subtype Identification

To investigate molecular heterogeneity among melanoma patients, unsupervised consensus clustering was performed on the TCGA‐SKCM cohort using the R package “ConsensusClusterPlus.” Clustering was conducted based on the gene expression matrix using the Euclidean distance metric and the k‐means clustering algorithm. To assess the robustness and stability of the clustering results, resampling was performed 1000 times. The number of clusters (*k*) was evaluated over a range from 2 to 9.

### 2.3 Dimensionality Reduction and Survival Analysis

To visually assess the separation among different patient subtypes, dimensionality reduction analyses were performed using t‐distributed stochastic neighbor embedding (t‐SNE) and Uniform Manifold Approximation and Projection (UMAP). OS was estimated using the Kaplan–Meier method, and survival differences among subtypes were compared using the log‐rank test. A two‐sided *p* value of < 0.05 was considered statistically significant.

### 2.4 Differential Expression Analysis and Gene Set Enrichment Analysis

Based on the consensus clustering results, differential expression analysis among distinct patient subtypes was performed using the “limma” package in R. Genes with an absolute log_2_ fold change (|log_2_FC|) > 1 and an adjusted *p* value of < 0.05 were considered differentially expressed. Differentially expressed genes were subsequently subjected to Gene Set Enrichment Analysis (GSEA) to systematically elucidate the underlying biological functions associated with each subtype. Reference gene sets included Gene Ontology (GO) and WikiPathways databases. The normalized enrichment score (NES) was used to evaluate pathway enrichment strength, with statistical significance defined as |NES| > 1 and a false discovery rate of < 0.25.

### 2.5 Tumor Immune Microenvironment and Multi‐Omics Analysis

Single‐sample gene set enrichment analysis (ssGSEA) was applied to quantify the relative infiltration levels of different immune cell subpopulations in the TCGA‐SKCM cohort. Immune‐related gene signatures were obtained from previously published studies and publicly available databases. The ESTIMATE algorithm was used to calculate the immune score, stromal score, and tumor purity for each sample, thereby enabling comprehensive evaluation of tumor microenvironment characteristics across different subtypes. In addition, multiple genomic and immunological features were integrated to comprehensively compare the multi‐omics profiles among subtypes, including tumor mutation burden, neoantigen load, T‐ and B‐cell receptor diversity (TCR/BCR Shannon index and richness), aneuploidy score, proliferation score, and wound‐healing signature score.

### 2.6 Weighted Gene Co‐Expression Network Analysis

Weighted gene co‐expression network analysis (WGCNA) was performed using the “WGCNA” package in R to construct gene co‐expression networks. To reduce noise from low‐variance genes, the top 25% of genes ranked by coefficient of variation were selected for network construction. The optimal soft‐thresholding power was determined based on the scale‐free topology criterion. Subsequently, a topological overlap matrix (TOM) was calculated, and hierarchical clustering was conducted based on the TOM. Modules were identified with a minimum module size of 30 genes, and highly similar modules were merged according to their correlation. Module–trait relationships were evaluated using Mantel tests and Pearson's correlation analyses to assess the associations between module eigengenes and patient subtypes as well as clinical phenotypes, including survival status and survival time. Modules significantly associated with the C3 subtype, which exhibited the poorest prognosis, were selected for subsequent analyses.

### 2.7 Hub Gene Identification and Risk Assessment Analysis

Within the target modules, hub genes were identified based on a combination of module membership (MM) and gene significance (GS). Univariate Cox proportional hazards regression analyses were subsequently performed across the TCGA cohort and multiple independent GEO data sets to estimate hazard ratios (HRs) and corresponding 95% confidence intervals for each candidate gene. Genes that consistently exhibited HR values > 1 with statistical significance across multiple independent cohorts were considered potential high‐risk driver genes.

### 2.8 Prediction and Screening of FOXQ1‐Related Target Genes

Potential downstream target genes regulated by FOXQ1 were predicted using the hTFtarget database, a comprehensive resource for human transcription factor–target gene interactions. Genes putatively regulated by FOXQ1 were retrieved based on experimentally supported and computationally inferred regulatory relationships provided by the database.

To further evaluate the clinical relevance of these predicted targets, differential expression analysis was performed using transcriptomic data from the TCGA Skin Cutaneous Melanoma (TCGA‐SKCM) cohort. FOXQ1‐related candidate genes were analyzed for differential expression between tumor and corresponding control samples (or between predefined expression groups, as applicable). Genes meeting the predefined criteria for differential expression were identified. Finally, the intersection between FOXQ1‐predicted target genes from the hTFtarget database and the differentially expressed genes in the TCGA‐SKCM data set was determined. A total of six overlapping genes were obtained and selected for subsequent analyses.

### 2.9 Clinical Specimens and Ethics Approval

Tumor tissues from patients with pathologically confirmed malignant melanoma (MM) and paired adjacent nontumor skin tissues (located at least 2 cm from the tumor margin) were collected. Following surgical resection, each specimen was divided into two parts: one portion was fixed in 4% paraformaldehyde for paraffin embedding and subsequent immunohistochemical analysis, while the other portion was immediately snap‐frozen in liquid nitrogen and stored at −80°C for RNA and protein extraction. The general inclusion criteria were as follows: (1) no prior radiotherapy, chemotherapy, or systemic targeted or immunotherapy before surgery; (2) histopathological confirmation of malignant melanoma; and (3) availability of complete clinical and follow‐up data. Patients with concomitant malignancies or severe systemic diseases were excluded. The sample size and detailed clinicopathological characteristics can be specified according to the actual study population. This study was approved by the Ethics Committee of the participating institution (Approval Number: 20221731). Written informed consent was obtained from all patients prior to surgery.

### 2.10 Cell Lines, Reagents, and Culture Conditions

Human melanoma cell lines (SK‐Mel‐19, SK‐Mel‐28, SK‐Mel‐94, and SK‐Mel‐103) and normal human epidermal melanocytes (NHM) were obtained from accredited biological resource centers. Upon receipt, all cell lines were authenticated and cultured according to the suppliers' instructions, and routine mycoplasma testing was performed to ensure the absence of contamination. Cells were maintained in Dulbecco's modified Eagle's medium (DMEM) supplemented with 10% fetal bovine serum (FBS) and 1% penicillin–streptomycin solution, and incubated at 37°C in a humidified atmosphere containing 5% CO_2_. When cell confluence reached approximately 80%, cells were detached using 0.25% trypsin–EDTA and passaged at a ratio of 1:3 to 1:5. All functional experiments were performed using cells in the logarithmic growth phase.

### 2.11 Construction of Lentiviral Vectors and Establishment of Stable Knockdown Cell Lines

#### 2.11.1 shRNA Design and Vector Construction

Short hairpin RNA (shRNA) sequences specifically targeting the coding regions of human FOXQ1 and SSBP2 were designed using online prediction tools. Sequences with high predicted interference efficiency and low off‐target potential were selected, synthesized, and cloned into lentiviral expression vectors. A nontargeting shRNA sequence was used as a negative control (sh‐NC). After sequence verification by Sanger sequencing, the recombinant lentiviral vectors were co‐transfected into HEK293T cells together with packaging plasmids (such as psPAX2 and pMD2.G). Viral supernatants were collected 48–72 h after transfection, filtered to remove cell debris, and used to obtain lentiviral particles. Viral titers were subsequently determined. The detailed shRNA sequences are provided in Table S1.

#### 2.11.2 Cell Infection and Selection

SK‐Mel‐94 and SK‐Mel‐103 cells were seeded into 6‐well plates and cultured until reaching approximately 30%–40% confluence. Lentiviral supernatants with different multiplicities of infection were then added to the cells, together with polybrene (4–8 μg/mL) to enhance infection efficiency. After 12–24 h of incubation, the medium was replaced with fresh complete culture medium, and cells were allowed to continue growing. At 48 h postinfection, antibiotic selection was initiated according to the resistance gene carried by the lentiviral vector (e.g., puromycin resistance). Cells were cultured under selective pressure for 5–7 days to eliminate noninfected cells, thereby generating stable cell populations with FOXQ1 or SSBP2 knockdown.

The knockdown efficiency of FOXQ1 and SSBP2 was evaluated at both the mRNA and protein levels using quantitative real‐time PCR (qRT‐PCR) and Western blot analysis, respectively. The shRNA construct exhibiting the highest knockdown efficiency was selected for subsequent experiments.

### 2.12 RNA Extraction, cDNA Synthesis, and Quantitative Real‐Time PCR

#### 2.12.1 Total RNA Extraction

Total RNA was extracted from tissues or cultured cells using TRIzol reagent according to the manufacturer's instructions. Briefly, an appropriate amount of tissue or cells was lysed in TRIzol reagent and incubated at room temperature for 5 min. Chloroform was then added, followed by vigorous shaking and centrifugation to allow phase separation. The aqueous phase was carefully collected and mixed with an equal volume of isopropanol to precipitate RNA. The RNA pellet was washed with 75% ethanol, air‐dried, and subsequently dissolved in RNase‐free water. RNA concentration and purity were determined using a NanoDrop spectrophotometer or an equivalent microvolume spectrophotometer, and only samples with an A260/A280 ratio between 1.8 and 2.0 were used for subsequent experiments.

#### 2.12.2 Reverse Transcription and qRT‐PCR

Complementary DNA (cDNA) was synthesized from total RNA using a reverse transcription kit according to the manufacturer's protocol. qRT‐PCR was performed using a SYBR Green‐based detection system on a real‐time PCR instrument. GAPDH was used as the internal reference gene, and relative gene expression levels were calculated using the 2^−ΔΔCt^ method. Primers for FOXQ1, SSBP2, GAPDH, and β‐actin were synthesized commercially. Primer specificity and amplification efficiency were validated by preliminary experiments. The sequences of the qRT‐PCR primers are listed in Table 1.

**Table 1:** Primer sequences used for qRT‐PCR.

Gene	Forward primer (5′–3′)	Reverse primer (5′–3′)	Host species
FOXQ1	CAGTTCCTGGACTTCCAGAA	TGCTGAGGTGGTTGTAGTTG	Human
SSBP2	GGAGACTTCCTGGAGTTCTG	TCCAGGTCTTCAGGAGGTTT	Human
GAPDH	GGAGCGAGATCCCTCCAAAAT	GGCTGTTGTCATACTTCTCATGG	Human

### 2.13 Protein Extraction and Western Blot Analysis

Treated cells or homogenized tissue samples were collected and lysed in prechilled RIPA lysis buffer supplemented with protease and phosphatase inhibitors. The samples were incubated on ice for 30 min with gentle pipetting at intervals to ensure complete lysis, followed by centrifugation at 12,000 *g* for 15 min at 4°C. The supernatants were collected for protein quantification. Protein concentrations were determined using the bicinchoninic acid assay. Equal amounts of protein (20–40 μg) were mixed with 5× SDS loading buffer and denatured by boiling at 95°C for 5 min. The samples were then separated by SDS–polyacrylamide gel electrophoresis (SDS–PAGE) and transferred onto polyvinylidene difluoride (PVDF) membranes. After transfer, the membranes were blocked with 5% (w/v) nonfat milk dissolved in Tris‐buffered saline containing 0.1% Tween‐20 (TBST) at room temperature for 1 h, and then incubated with primary antibodies (e.g., FOXQ1, SSBP2, and β‐actin) overnight at 4°C. On the following day, the membranes were washed three times with TBST (5–10 min each) and incubated with horseradish peroxidase (HRP)‐conjugated secondary antibodies at room temperature for 1 h. After additional washes with TBST, protein bands were visualized using enhanced chemiluminescence reagents and captured with a gel imaging system. Band intensities were quantified using ImageJ software. The expression levels of target proteins were normalized to GAPDH and subjected to statistical analysis. Detailed information on the primary and secondary antibodies used in this study is provided in Table 2.

**Table 2:** Primary and secondary antibodies.

Antibody	Supplier	Catalog number	Host species
FOXQ1	Abcam	ab51340	Rabbit
SSBP2	Abcam	ab184186	Rabbit
GAPDH	Proteintech	60004‐1‐Ig	Mouse
β‐Actin	Proteintech	66009‐1‐Ig	Mouse
HRP‐goat anti‐rabbit IgG	Proteintech	SA00001‐2	Goat
HRP‐goat anti‐mouse IgG	Proteintech	SA00001‐1	Goat

### 2.14 Cell Proliferation Assays

#### 2.14.1 Cell Counting Kit‐8 (CCK‐8) Cell Viability Assay

Cells from the control group and each treatment group were harvested by trypsinization and prepared as single‐cell suspensions. After cell counting, the cell density was adjusted, and cells were seeded into 96‐well plates at a density of approximately 2 × 10^3^ to 5 × 10^3^ cells per well, with six replicate wells for each group. After cell attachment, Cell Counting Kit‐8 (CCK‐8) reagent (10 μL per well) was added at 0, 24, 48, and 72 h (96 h was included when necessary), followed by incubation at 37°C for 1–2 h. The absorbance was measured at 450 nm (OD_450_) using a microplate reader. Cell growth curves were generated, and changes in OD values at different time points were compared among groups to evaluate the effects of FOXQ1 knockdown, SSBP2 knockdown, and their combined intervention on the proliferative capacity of melanoma cells.

#### 2.14.2 Colony Formation Assay

Treated cells were digested into single‐cell suspensions and seeded into 6‐well plates or culture dishes at a density of approximately 500–1000 cells per well. The plates were gently agitated to ensure even cell distribution and incubated at 37°C in a humidified atmosphere containing 5% CO_2_ for 10–14 days. During the incubation period, the culture medium was replaced every 2–3 days until visible colonies formed, defined as clusters containing ≥ 50 cells. At the end of the incubation, cells were gently washed twice with phosphate‐buffered saline (PBS) and fixed with 4% paraformaldehyde at room temperature for 15–20 min. After fixation, the cells were stained with 0.1% crystal violet for 15–30 min, rinsed with running water to remove excess dye, and air‐dried. Colonies were counted either under a microscope or by visual inspection. The colony formation rate was calculated using the following formula:

Colony formation rate (%) = (Number of colonies/Number of seeded cells) × 100%.

### 2.15 Cell Migration and Invasion Assays

#### 2.15.1 Wound Healing Assay

Cells were seeded into 6‐well plates and cultured until reaching approximately 80%–90% confluence. A sterile 200‐μL pipette tip was then used to generate a straight scratch across the cell monolayer. The supernatant was removed, and the wells were gently washed 1–2 times with PBS to eliminate detached cells. Serum‐free or low‐serum culture medium was subsequently added to minimize the influence of cell proliferation. Images of the same fields were captured at 0 and 48 h using an inverted microscope, and the wound width was recorded. Wound areas or widths were measured using ImageJ software, and the migration rate was calculated as follows:

Migration rate (%) = (Initial wound width − Final wound width)/Initial wound width × 100%.

#### 2.15.2 Transwell Invasion Assay

Transwell chambers equipped with polycarbonate membranes containing 8‐μm pores were used for the invasion assay. The upper surface of the membrane was precoated with an appropriate amount of diluted Matrigel (prepared according to the manufacturer's instructions) and incubated at 37°C for 30–60 min to allow gel formation. After treatment, cells were resuspended in serum‐free medium and counted to adjust the cell concentration (e.g., 2 × 10^5^ to 5 × 10^5^ cells/mL). A defined volume of the cell suspension was added to the upper chamber, while the lower chamber was filled with medium containing 10% FBS as a chemoattractant. The chambers were incubated at 37°C in a humidified atmosphere with 5% CO_2_ for 24 h. Following incubation, noninvaded cells on the upper surface of the membrane were gently removed using a cotton swab. The membranes were then washed with PBS, fixed with 4% paraformaldehyde at room temperature for 15–20 min, and stained with 0.1% crystal violet for 15–30 min. Cells that had invaded through the Matrigel and adhered to the lower surface of the membrane were counted in multiple randomly selected high‐power fields under a light microscope to assess invasive capacity.

### 2.16 TUNEL Staining Assay

Cells were seeded into 24‐well plates containing precoated glass coverslips. After treatment according to the experimental grouping, cells were fixed with 4% paraformaldehyde and permeabilized with 0.1% Triton X‐100 at room temperature for 10–15 min. Following washing with PBS, the TUNEL reaction mixture was prepared according to the manufacturer's instructions and applied to the samples, which were then incubated at 37°C for 1 h in the dark. After incubation, the samples were washed with PBS and counterstained with 4′,6‐diamidino‐2‐phenylindole to visualize cell nuclei. Apoptotic cells were observed and imaged using a fluorescence microscope. Multiple random fields were selected for quantification, and the apoptotic rate was calculated as the percentage of TUNEL‐positive cells relative to the total number of cells. Apoptosis levels were compared among different treatment groups.

### 2.17 Chromatin Immunoprecipitation (ChIP) and Dual‐Luciferase Reporter Assays

#### 2.17.1 Chromatin Immunoprecipitation (ChIP) Assay

SK‐Mel‐103 cells, which exhibit relatively high FOXQ1 expression, were selected for the ChIP assay. According to the manufacturer's protocol, cells were cross‐linked with 1% formaldehyde at room temperature for 10 min, and the cross‐linking reaction was terminated by adding a quenching solution. Cells were then collected and lysed to extract chromatin. Chromatin was sheared by sonication to generate DNA fragments of approximately 200–1000 bp. A portion of the sheared chromatin was reserved as the input control, while the remaining chromatin was incubated with a FOXQ1‐specific antibody or an equal amount of normal IgG as a negative control, followed by immunoprecipitation using Protein A/G magnetic beads. After extensive washing to remove nonspecific interactions, cross‐links were reversed, and samples were treated with proteinase K. The DNA was then purified and subjected to quantitative real‐time PCR (qPCR) using primers specific to the SSBP2 promoter region. Enrichment levels were normalized to the input control, and the fold enrichment of the SSBP2 promoter in the FOXQ1 antibody group was compared with that in the IgG control group to evaluate the binding ability of FOXQ1 to the SSBP2 promoter. The sequences of the ChIP primers are listed in Table 3.

**Table 3:** ChIP primer sequences for SSBP2 promoter region.

Gene	Primer sequence (forward)	Primer sequence (reverse)
SSBP2	ATGCTTTGAGTAGGGTC	TTGTTGTGTGTGTTTTG

#### 2.17.2 Dual‐Luciferase Reporter Assay

Based on ChIP prediction results and transcription factor binding site analysis, DNA fragments containing the key regulatory region of the SSBP2 promoter were amplified and cloned into a luciferase reporter vector to generate the wild‐type (WT) SSBP2 promoter construct. Site‐directed mutagenesis was performed to disrupt the FOXQ1 binding site, thereby generating the corresponding mutant (MUT) construct. The WT or MUT reporter plasmids were co‐transfected into SK‐Mel‐103 cells together with FOXQ1 shRNA or the corresponding control plasmid. A Renilla luciferase plasmid was simultaneously co‐transfected as an internal control. After 24–48 h of transfection, firefly and Renilla luciferase activities were measured using a dual‐luciferase reporter assay system according to the manufacturer's instructions. Relative luciferase activity was calculated by normalizing firefly luciferase activity to Renilla luciferase activity. Differences in luciferase activity among groups were compared to verify the regulatory effect of FOXQ1 on the transcriptional activity of the SSBP2 promoter.

### 2.18 Xenograft Tumor Model and In Vivo Experiments

Male BALB/c nude mice aged 4–6 weeks were obtained and acclimatized for 1 week in a specific pathogen‐free animal facility, with free access to food and water. Animals were housed under controlled conditions of constant temperature and humidity with a 12 h light/dark cycle throughout the experiment. SK‐Mel‐103 cells with stable FOXQ1 knockdown, or cells with simultaneous knockdown of FOXQ1 and SSBP2, were harvested, counted, and resuspended in sterile PBS or a Matrigel:PBS mixture. The cell concentration was adjusted to approximately 5 × 10^6^ cells per 100 μL. Each mouse was subcutaneously injected with 100 μL of the cell suspension into the right axilla or dorsal flank. Mice were randomly divided into groups, including the sh‐NC group, sh‐FOXQ1 group, sh‐FOXQ1 + sh‐NC group, and sh‐FOXQ1 + sh‐SSBP2 group, with six mice per group. Tumor length and width were measured every 3–4 days using digital calipers, and tumor volume was calculated using the following formula:

Tumor volume = 1/2 × (length × width^2^).

Tumor growth curves were recorded. At the experimental endpoint (e.g., 4 weeks after injection or when tumor volume reached the predetermined limit), mice were euthanized, and tumors were excised and weighed. Final tumor volume and tumor weight were recorded for each group. All animal experiments were approved by the Institutional Animal Care and Use Committee of the corresponding institution (Approval Number: 20221731), and all procedures were performed in accordance with institutional guidelines and regulations for animal welfare.

### 2.19 Immunohistochemistry (IHC) Staining

Mouse tumor tissues and selected clinical specimens were fixed in 4% paraformaldehyde, followed by routine dehydration, paraffin embedding, and sectioning at a thickness of approximately 4 μm. Tissue sections were deparaffinized in xylene and rehydrated through a graded ethanol series, then subjected to antigen retrieval by microwave heating in citrate buffer. After cooling to room temperature, endogenous peroxidase activity was blocked by incubation with 3% hydrogen peroxide for 10–15 min. Sections were washed with PBS and blocked with normal serum at room temperature for 20–30 min. After removal of the blocking solution, sections were incubated with primary antibodies (e.g., anti‐SSBP2 antibody) overnight at 4°C. On the following day, sections were brought to room temperature, washed with PBS, and incubated with appropriate secondary antibodies. Immunoreactivity was visualized using 3,3′‐diaminobenzidine as the chromogen. The color development was monitored under a light microscope and terminated by rinsing with distilled water. Nuclei were counterstained with hematoxylin, followed by dehydration, clearing, and mounting. For semi‐quantitative analysis, multiple random fields were selected under a light microscope. Staining intensity (scored as 0–3) and the percentage of positive cells (scored as 0–4) were evaluated, and the immunohistochemical (IHC) score was calculated by multiplying the two scores. Differences in the expression levels of SSBP2 and other target proteins among groups were subsequently compared.

### 2.20 Statistical Analysis

All experiments were independently repeated at least three times. Data are presented as the mean ± standard deviation. Comparisons between two groups were performed using Student's *t*‐test, while comparisons among multiple groups were conducted using one‐way analysis of variance (ANOVA), followed by post hoc multiple comparison tests when appropriate. Categorical data were analyzed using the *χ* ^2^ test or Fisher's exact test. Pearson's correlation analysis was applied to assess correlations between variables. All statistical analyses were performed using GraphPad Prism software. A two‐tailed *p* < 0.05 was considered statistically significant.

## 3 Results

### 3.1 Identification of Molecular Subtypes of Melanoma Based on Consensus Clustering and Analysis of Prognostic Differences

To explore the molecular heterogeneity of the melanoma patient cohort, consensus clustering analysis was performed based on the TCGA transcriptomic profiles. The consensus cumulative distribution function (CDF) curves (Figure 1A) demonstrated a progressive increase in clustering stability as the number of clusters (*k*) increased, with the curves transitioning from a steep shape at *k* = 2 to a relatively flat plateau at *k* = 9. The delta area plot (Figure 1B) further indicated that the marginal gain in clustering stability markedly decreased after *k* = 3, suggesting that *k* = 3 represented the optimal number of clusters for this cohort. The consensus matrix heatmap revealed that when *k* = 3, samples within the same cluster exhibited consensus indices approaching 1, whereas samples from different clusters showed relatively low consensus values, confirming the robustness and reliability of the three identified molecular subtypes (Figure 1C). In addition, the cluster‐consensus bar plot and the proportion of ambiguous clustering (PAC) analysis (Figure 1D,E) demonstrated that the median consensus scores of all three clusters were close to 1, indicating high intracluster homogeneity and further supporting *k* = 3 as the optimal clustering solution. Dimensionality reduction and visualization using t‐SNE (Figure 1F) and UMAP (Figure 1G) revealed a clear separation among the three molecular subtypes in low‐dimensional space, providing additional validation of the consensus clustering results. To assess the clinical relevance of these molecular subtypes, Kaplan–Meier survival analysis was conducted. The results showed a significant difference in OS among the three subtypes (log‐rank test, *p* < 0.001). Among them, patients in the CS3 subtype exhibited the poorest prognosis, with a median OS of approximately 2.5 years, whereas patients in the CS2 subtype had the most favorable prognosis, with a median OS of approximately 41.0 years (Figure 1H). These findings suggest that consensus clustering–based molecular subtyping serves as a robust and independent prognostic stratification indicator for this melanoma cohort.

**Figure 1:**
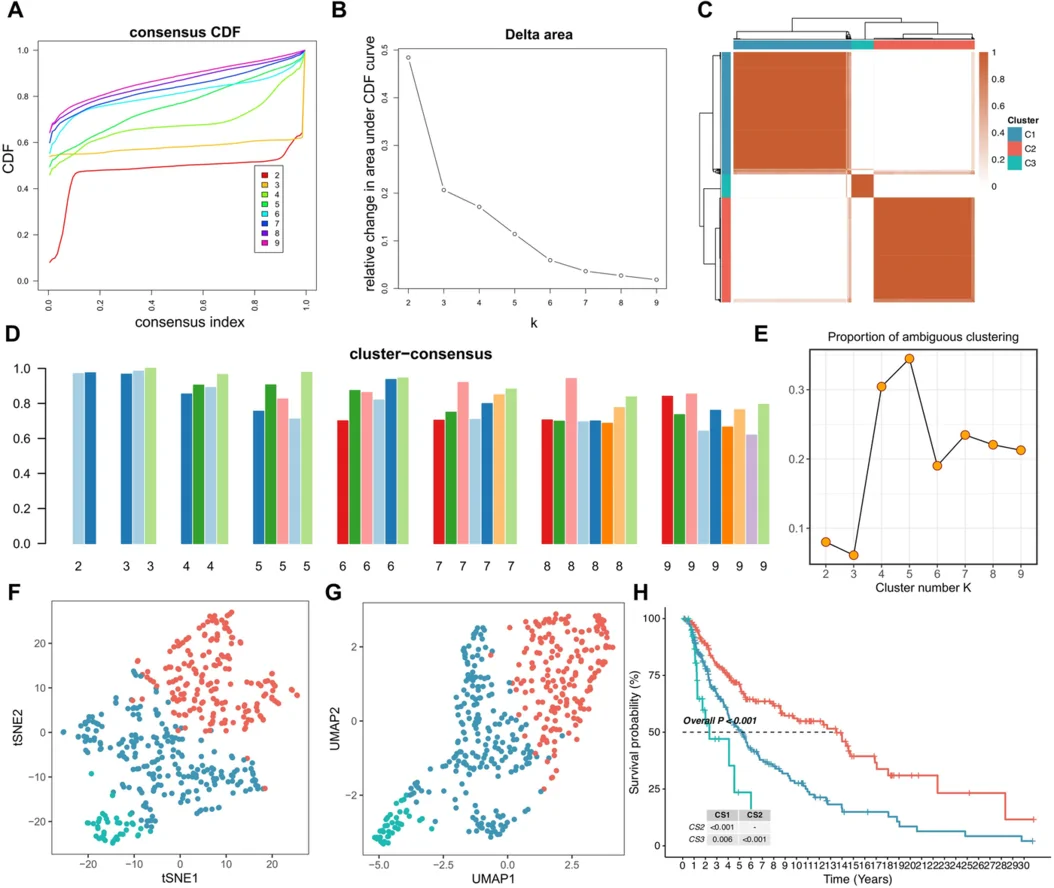
Identification of molecular subtypes of melanoma based on consensus clustering and prognostic analysis. (A) Consensus cumulative distribution function (CDF) curves for different numbers of clusters (*k* = 2–9). (B) Delta area plot showing changes in the area under the CDF curves for different *k* values. (C) Consensus matrix heatmap at *k* = 3. (D) Bar plot showing the median cluster‐consensus scores for different numbers of clusters (*k* = 2–9). (E) Proportion of ambiguous clustering (PAC) across different *k* values. (F) t‐distributed stochastic neighbor embedding (t‐SNE) visualization of the three molecular subtypes. (G) Uniform Manifold Approximation and Projection (UMAP) visualization of the three molecular subtypes. (H) Kaplan–Meier overall survival (OS) curves for the three molecular subtypes (C1, C2, and C3).

### 3.2 Transcriptional Characteristics, Functional Enrichment, and Signaling Pathway Differences Among Consensus Clustering–Based Melanoma Molecular Subtypes

To characterize the molecular and functional features of the three consensus clustering–derived melanoma subtypes (C1, C2, and C3), we first analyzed the expression patterns of key transcription factors and signaling pathway–related genes. Heatmap visualization of Z‐score–normalized expression profiles revealed distinct transcriptional signatures across the three subtypes, indicating marked molecular heterogeneity among C1, C2, and C3 (Figure 2A). Next, GSEA was performed to identify subtype‐specific biological pathways and to explore their associations with tumor‐related phenotypes. GO functional enrichment heatmaps illustrated the biological processes enriched in each subtype, with NES used to quantify pathway activation levels. The C3 subtype exhibited significantly higher NES values than C1 and C2 across multiple tumor progression–related pathways, suggesting enhanced malignant potential and a greater propensity for invasive growth and metastasis. In contrast, the C1 subtype showed higher NES values in immune response–associated pathways, including cytotoxic T‐cell activity and antigen presentation processes, indicating a more active immune surveillance and antitumor immune response, which may contribute to improved tumor control (Figure 2B). The C2 subtype was predominantly enriched in pathways related to metabolism, tumor suppression, and cellular repair, particularly those involved in energy metabolism and tumor‐inhibitory mechanisms. These molecular characteristics likely confer enhanced metabolic regulation and growth restraint, which may explain the favorable survival outcomes observed in the C2 subtype. Conversely, the C3 subtype displayed pronounced dysregulation in pathways related to DNA damage repair and chromosome segregation, with consistently negative enrichment levels, suggesting increased genomic instability, susceptibility to malignant transformation, and a higher likelihood of chromosomal aberrations (Figure 2B). Hallmark pathway enrichment analysis further demonstrated that the C2 subtype was significantly enriched in immune and inflammatory signaling pathways, such as interferon‐α/γ responses and TNF‐α signaling via NF‐κB. In contrast, the C3 subtype showed preferential enrichment in pathways associated with cell proliferation, metabolic reprogramming, and MYC‐ and E2F‐target gene signaling, which are closely linked to tumor growth and progression (Figure 2D). Differential expression analysis visualized by volcano plots revealed that immune‐ and inflammation‐related genes, including SPI1, MEF2C, NFKB1, and RELA, were significantly upregulated in the C2 subtype. In contrast, the C3 subtype showed marked upregulation of transcription factors such as ELF3, PRDM14, TP63, EHF, TFAP2C, HBP1, and CDX2, which are involved in epithelial differentiation, stem cell fate determination, embryonic development, and tumor progression. The coordinated upregulation of these factors suggests that C3 tumors are characterized by enhanced epithelial differentiation programs, sustained stemness, and aggressive malignant behavior (Figure 2E), thereby highlighting potential key drivers for subsequent mechanistic investigation.

**Figure 2:**
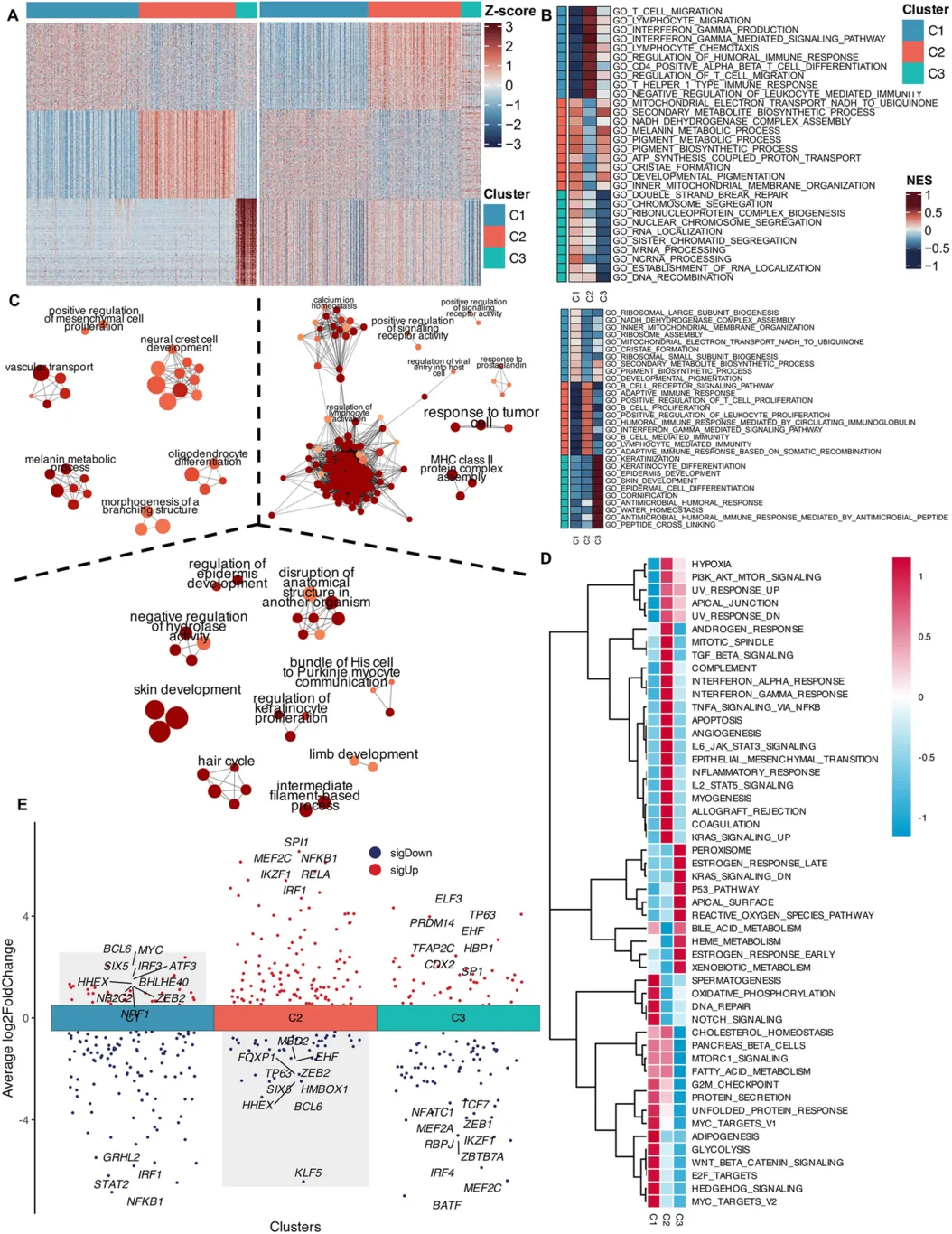
Transcriptional characteristics, functional enrichment, and signaling pathway differences among consensus clustering–based melanoma molecular subtypes. (A) Heatmap of transcriptomic expression profiles in melanoma samples across different molecular subtypes (C1, C2, and C3). (B) Heatmap of Gene Ontology (GO) Biological Process enrichment results based on gene set enrichment analysis (GSEA) among different molecular subtypes. (C) Functional module network of differentially expressed genes. (D) Differential enrichment analysis of Hallmark signaling pathways across the molecular subtypes. (E) Distribution of expression changes of representative transcription factors across different molecular subtypes.

### 3.3 Immune Microenvironment Characteristics and Multi‐Omics Differentiation of Consensus Clustering–Based Melanoma Subtypes

Next, we investigated differences in the tumor immune microenvironment among the three consensus clustering–derived subtypes (C1, C2, and C3), with a particular focus on immune cell infiltration, immune‐related scores, stromal components, and tumor purity. This analysis aimed to elucidate the role of the immune microenvironment in subtype‐specific tumor biology and its potential impact on tumor initiation, progression, and therapeutic response. A heatmap of immune cell infiltration illustrated distinct infiltration patterns across the three subtypes (Figure 3A). Quantitative analysis using box plots further revealed significant differences in immune scores among the clusters, reflecting the overall activity of the immune system within the tumor microenvironment. Immune scores were calculated based on the quantified levels of immune cell infiltration. Notably, the C2 subtype exhibited significantly higher immune scores than the C1 and C3 subtypes, indicating a highly immune‐activated tumor microenvironment. This elevated immune score in C2 was associated with active immune cell infiltration and enhanced immune responses, suggesting that tumors of this subtype may exhibit greater sensitivity to immunotherapy. In contrast, the C1 subtype displayed the lowest immune scores, indicative of a predominantly immunosuppressive tumor microenvironment. Such an environment may facilitate immune evasion by limiting effective antitumor immune responses, thereby promoting malignant progression. The immune score of the C3 subtype was intermediate between those of C1 and C2, suggesting a moderately active immune microenvironment that lacks both the strong immune activation observed in C2 and the pronounced immunosuppression characteristic of C1. In addition, differences in stromal scores and tumor purity were observed among the three subtypes. The C2 subtype exhibited higher stromal scores and lower tumor purity, implying a tumor microenvironment enriched with immune and stromal components. Collectively, these findings indicate that immune microenvironmental heterogeneity among the C1, C2, and C3 subtypes represents a critical contributor to melanoma heterogeneity and clinical outcome. The pronounced immune activation observed in the C2 subtype may underlie its superior prognosis and enhanced responsiveness to immunotherapeutic strategies (Figure 3B). To further characterize immune‐related molecular differences, correlation analysis based on Z‐score–normalized expression levels was performed. Heatmap visualization of immune checkpoint molecules and immune functional genes (red indicating high expression and blue indicating low expression) revealed marked divergence in immune molecular expression patterns across subtypes. The C2 subtype displayed widespread upregulation of immune‐related molecules, including antigen presentation–related genes (e.g., HLA family members), co‐stimulatory and co‐inhibitory molecules (e.g., ICOSLG, PDCD1LG2), and cytokines (e.g., IFNG, IL12), indicating a globally activated immune signaling network. In contrast, most immune‐related molecules in the C1 and C3 subtypes were significantly downregulated, with only a limited number of receptor–ligand pairs showing moderate expression, reflecting an overall immunologically suppressed state. The elevated expression of antigen presentation machinery in the C2 subtype further supports its enhanced immunogenicity, while the concurrent upregulation of co‐stimulatory and co‐inhibitory molecules highlights an active but tightly regulated immune microenvironment. Taken together, these findings classify C2 as an immune‐active subtype, whereas C1 and C3 represent immune‐suppressed subtypes, providing a molecular basis for immune‐based stratification in melanoma treatment (Figure 3C). Multi‐omics clustering heatmaps further revealed distinct biological characteristics among the subtypes. The C1 subtype exhibited features of high genomic instability, with significant enrichment in homologous recombination deficiency, aneuploidy score, fraction of genome altered, and number of genomic segments. In addition, both silent and nonsilent mutation rates, as well as proliferation and wound‐healing signature scores, were elevated, suggesting increased genomic damage and proliferative capacity. These characteristics imply that the C1 subtype may be particularly sensitive to DNA damage–targeting therapies. In contrast, the C2 subtype demonstrated robust immune activity, characterized by significantly higher T‐cell and B‐cell receptor repertoire diversity metrics (TCR/BCR Shannon index and richness) compared with the C1 and C3 subtypes. Moreover, this subtype showed marked enrichment of insertion/deletion–derived neoantigens (Indel neoantigens), single‐nucleotide variant–derived neoantigens (SNV neoantigens), intratumor heterogeneity, and lymphocyte infiltration signature scores, indicating enhanced tumor immunogenicity and active immune cell infiltration. These features provide a strong rationale for the effectiveness of immunotherapy in C2 tumors. The C3 subtype, however, exhibited global downregulation across nearly all evaluated parameters. Immune‐related indicators, including TCR/BCR diversity metrics (evenness, Shannon index, and richness), neoantigen burden, intratumor heterogeneity, and lymphocyte infiltration scores, were all suppressed. Simultaneously, genomic instability, mutation burden, proliferation, and wound‐healing signatures were also reduced. This combined phenotype suggests that C3 represents an inert subtype characterized by both low malignant potential and low immune activity. Consequently, tumors of this subtype may respond poorly to immunotherapy and may also exhibit limited sensitivity to cytotoxic chemotherapy, indicating an unfavorable therapeutic profile (Figure 3D).

**Figure 3:**
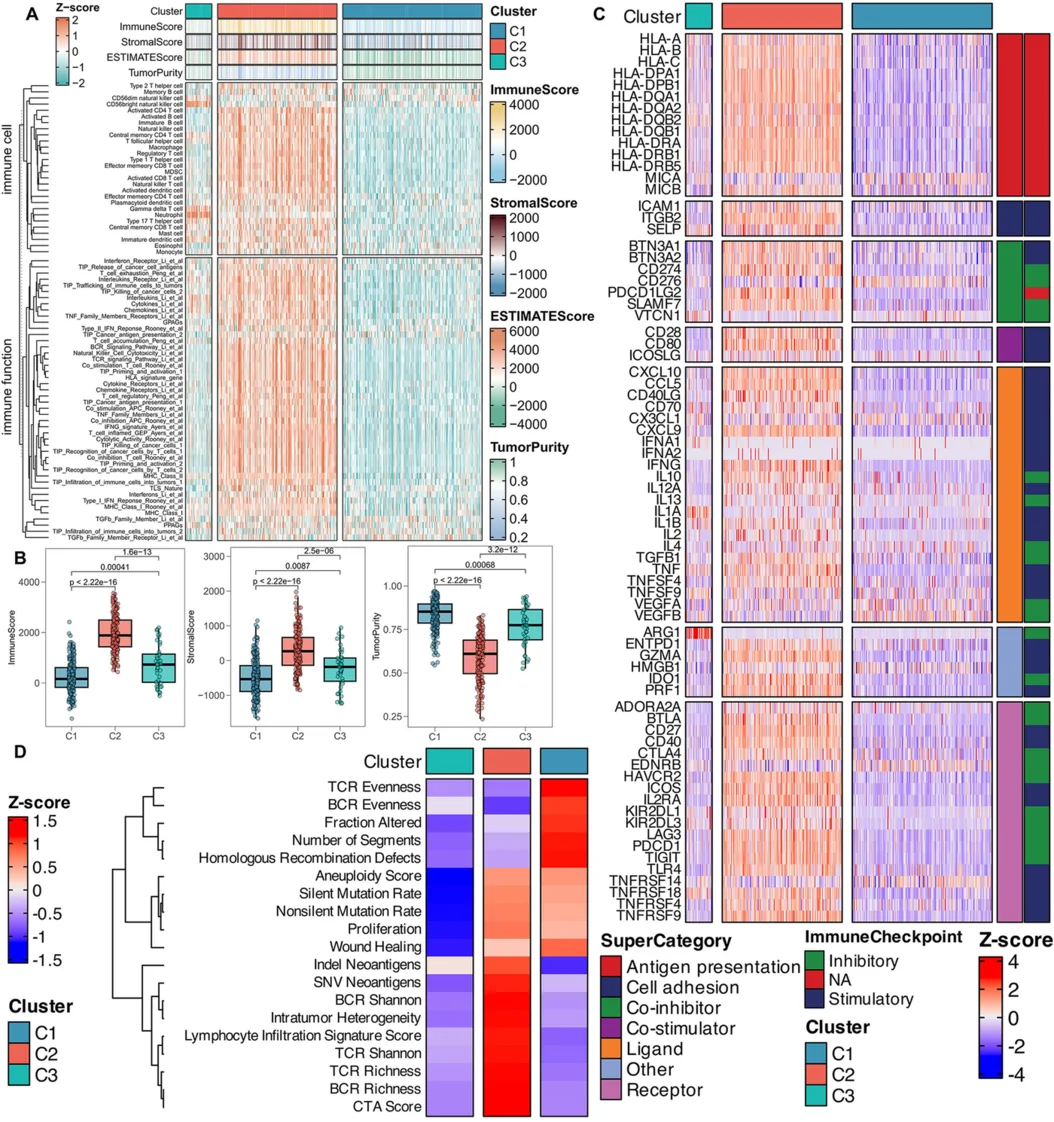
Multi‐omics characterization and immune‐based differentiation of consensus clustering–derived melanoma subtypes. (A) Heatmap of tumor immune microenvironment features across different molecular subtypes (C1, C2, and C3), evaluated using single‐sample gene set enrichment analysis (ssGSEA) and the ESTIMATE algorithm. (B) Box plots showing quantitative comparisons of immune score, stromal score, and tumor purity among different molecular subtypes. (C) Heatmap analysis of immune‐related molecular expression profiles across different molecular subtypes. (D) Integrated heatmap illustrating multi‐omics immune and genomic features across different molecular subtypes.

### 3.4 Phenotypic Association, Functional Characterization, and Prognostic Significance of Gene Co‐Expression Modules

In this study, WGCNA was applied to tumor transcriptomic data to identify multiple gene co‐expression modules (modules, MEs). By integrating multidimensional phenotype associations, functional enrichment analyses, and validation across clinical cohorts, we systematically characterized the biological functions and clinical relevance of these modules. A module–phenotype association network illustrated the correlation strength (Mantel's *r*) and statistical significance (Mantel's *P*) between the poorest‐prognosis subtype (C3), fusion status (fustat), and the identified gene modules (ME0, ME3, ME1, ME4, ME2, and ME5). The results demonstrated that the C3 subtype exhibited the strongest association with the ME3 module, reaching statistical significance at the 0.01 level. This finding indicates that ME3 serves as a molecular signature of the C3 subtype. Notably, the association between ME3 and fustat was weak and not statistically significant, suggesting that the molecular characteristics defining the C3 subtype are largely independent of survival outcome and that subtype identity and prognostic risk represent relatively distinct biological dimensions (Figure 4A). To elucidate the biological functions of the target gene modules, functional enrichment analyses were performed using the GO, Reactome (R‐HSA), and WikiPathways (WP) databases. Visualization of enrichment results revealed that the core biological functions of the ME3 module were predominantly enriched in pathways including GO:0008544, GO:0018149, and R‐HSA‐9725554, which correspond to epidermis development, peptide cross‐linking, and differentiation of interfollicular keratinocytes in mammalian skin, respectively. These pathways collectively emphasize roles in epithelial cell differentiation, skin tissue development, and structural maintenance (Figure 4B). Pearson's correlation analysis demonstrated a strong linear relationship between individual gene module membership and ME3 eigengene expression (*R* = 0.97, *p* < 2.2 × 10^−16^), with no evident outliers. This result indicates a highly cohesive co‐expression structure within the ME3 module, supporting its robustness for hub gene identification and cross‐cohort validation (Figure 4C). Consistently, bar plots revealed pronounced expression differences of differentially expressed genes within the ME3 module (Figure 4D,E). Subsequently, HR analyses were conducted across multiple melanoma‐related data sets, including GSE100797, GSE53118, GSE54467, and TCGA. The results consistently showed that FOXQ1 exhibited HR values > 1 in all four data sets, indicating that elevated FOXQ1 expression is significantly associated with poor prognosis and reduced survival in melanoma patients. These findings highlight FOXQ1 as a critical driver gene in melanoma progression and an important contributor to adverse clinical outcomes (Figure 4F).

**Figure 4:**
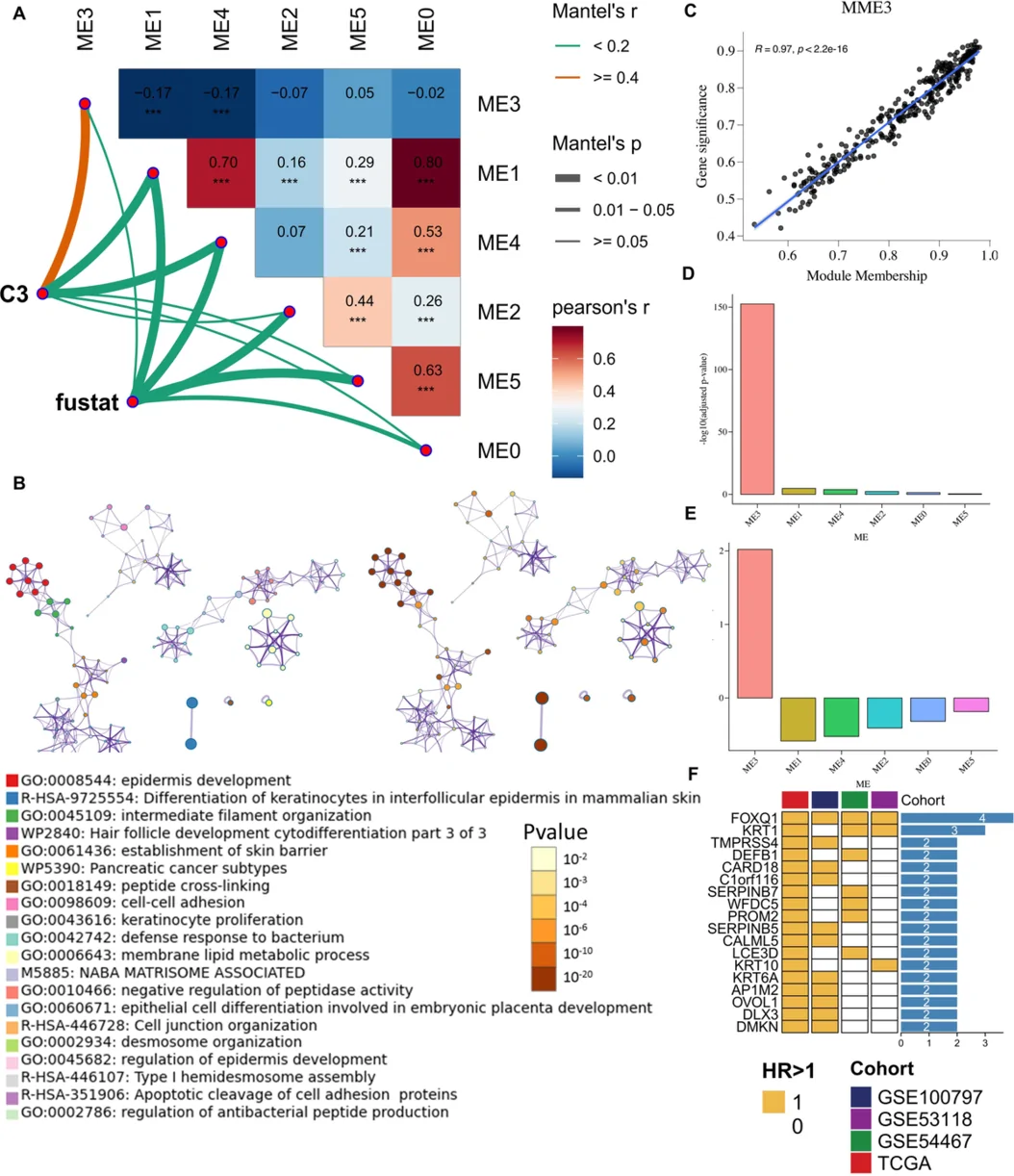
Phenotypic association, functional characterization, and prognostic analysis of gene co‐expression modules. (A) Module–phenotype association heatmap and network constructed based on weighted gene co‐expression network analysis (WGCNA), ***p < 0.001. (B) Functional enrichment network of the ME3 module. (C) Pearson's correlation analysis between module membership (MM) and gene significance (GS) for genes within the ME3 module. (D) Distribution of the significance of differentially expressed genes across distinct co‐expression modules. (E) Comparative analysis of overall expression change trends of differentially expressed genes among co‐expression modules. (F) Consistency analysis of hazard ratios (HRs) for candidate hub genes from the ME3 module across multiple independent melanoma cohorts.

### 3.5 Differential Expression of FOXQ1 in Malignant Melanoma

To investigate the expression pattern of FOXQ1 in malignant melanoma (MM), qRT‐PCR and Western blot (WB) analyses were performed using tumor tissues from MM patients and nonmalignant melanocytic tissues. The results demonstrated that both FOXQ1 mRNA and protein expression levels were significantly elevated in MM tissues compared with noncancerous melanocytic tissues (Figure 5A,B). To further explore the potential role of FOXQ1 in MM progression in vitro, we examined FOXQ1 expression across multiple MM cell lines (SK‐Mel‐19, SK‐Mel‐28, SK‐Mel‐94, and SK‐Mel‐103) and normal human melanocytes (NHM). Consistent with the clinical tissue findings, FOXQ1 expression was markedly upregulated at both the mRNA and protein levels in all MM cell lines relative to NHM cells (Figure 5C,D). Collectively, these results indicate that FOXQ1 is aberrantly overexpressed in malignant melanoma. However, whether FOXQ1 actively contributes to MM progression and its underlying molecular mechanisms remain to be further elucidated.

**Figure 5:**
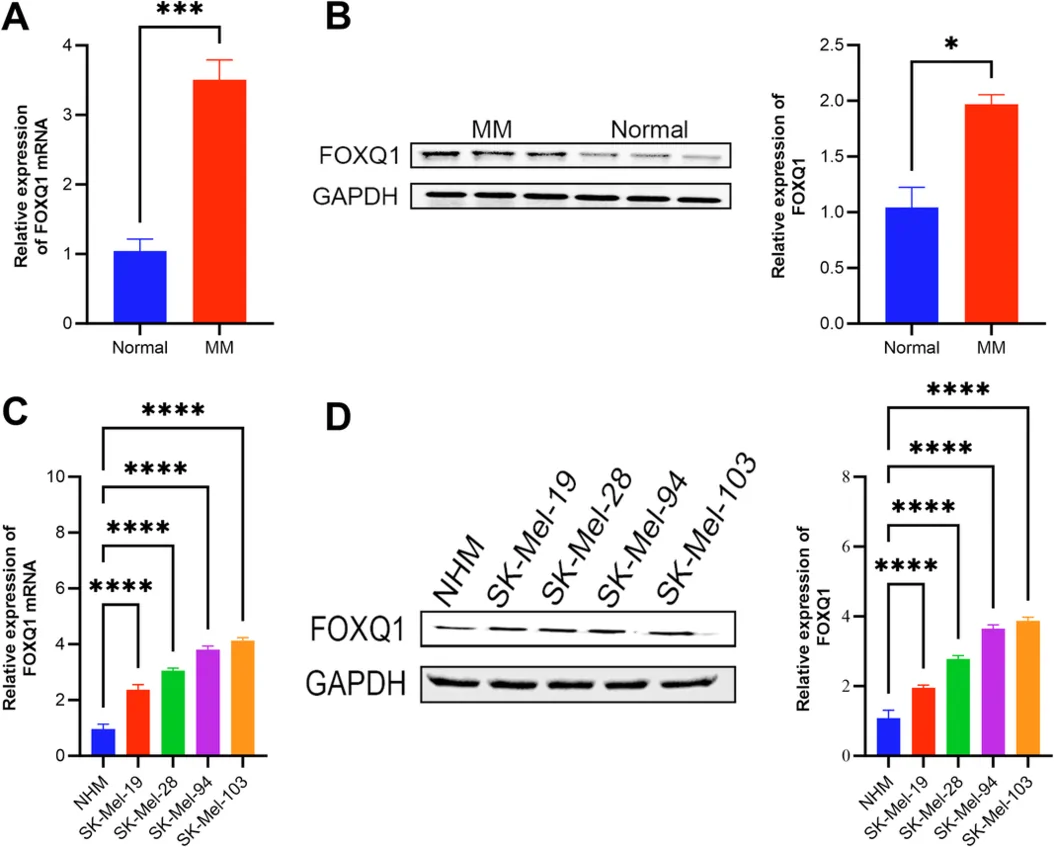
Differential expression of FOXQ1 in malignant melanoma. (A) FOXQ1 mRNA expression in melanoma and nonmalignant melanocytic tissues detected by quantitative real‐time PCR (qRT‐PCR). (B) FOXQ1 protein expression in melanoma and nonmalignant melanocytic tissues detected by Western blot analysis. (C) FOXQ1 mRNA expression in four melanoma cell lines (SK‐Mel‐19, SK‐Mel‐28, SK‐Mel‐94, and SK‐Mel‐103) and normal human melanocytes (NHM) measured by qRT‐PCR. (D) FOXQ1 protein expression in melanoma cell lines and NHM cells assessed by Western blot analysis (*p < 0.05; ***p < 0.001; ****p < 0.0001, *n* = 3).

### 3.6 Silencing FOXQ1 Suppresses Malignant Behaviors of Melanoma Cells in SK‐Mel‐94 and SK‐Mel‐103 Cell Lines

Based on the aforementioned findings, SK‐Mel‐94 and SK‐Mel‐103 cell lines, which exhibited the highest FOXQ1 expression levels among the tested melanoma cell lines, were selected for in vitro functional assays. FOXQ1 expression was silenced in these cells via lentiviral transduction. qRT‐PCR analysis confirmed a significant reduction in FOXQ1 mRNA expression in both cell lines following transduction (Figure 6A). Consistently, Western blot analysis demonstrated a marked decrease in FOXQ1 protein levels, indicating successful knockdown of FOXQ1 (Figure 6B). To evaluate the functional impact of FOXQ1 on melanoma malignancy, a series of assays was performed to assess cell proliferation, migration, and apoptosis. The CCK‐8 assay revealed that FOXQ1 silencing significantly suppressed the proliferative capacity of both SK‐Mel‐94 and SK‐Mel‐103 cells (Figure 6C). Wound healing assays further showed that FOXQ1 knockdown markedly reduced the migratory ability of these melanoma cells (Figure 6D). Moreover, TUNEL staining demonstrated a significant increase in the number of TUNEL‐positive cells in both SK‐Mel‐94 and SK‐Mel‐103 cell lines following FOXQ1 silencing, indicating enhanced apoptotic activity (Figure 6E). Collectively, these results demonstrate that FOXQ1 plays a critical role in regulating melanoma cell proliferation, migration, and apoptosis. Silencing FOXQ1 effectively attenuates malignant phenotypes of melanoma cells and promotes tumor cell apoptosis, highlighting FOXQ1 as a potential therapeutic target in malignant melanoma.

**Figure 6:**
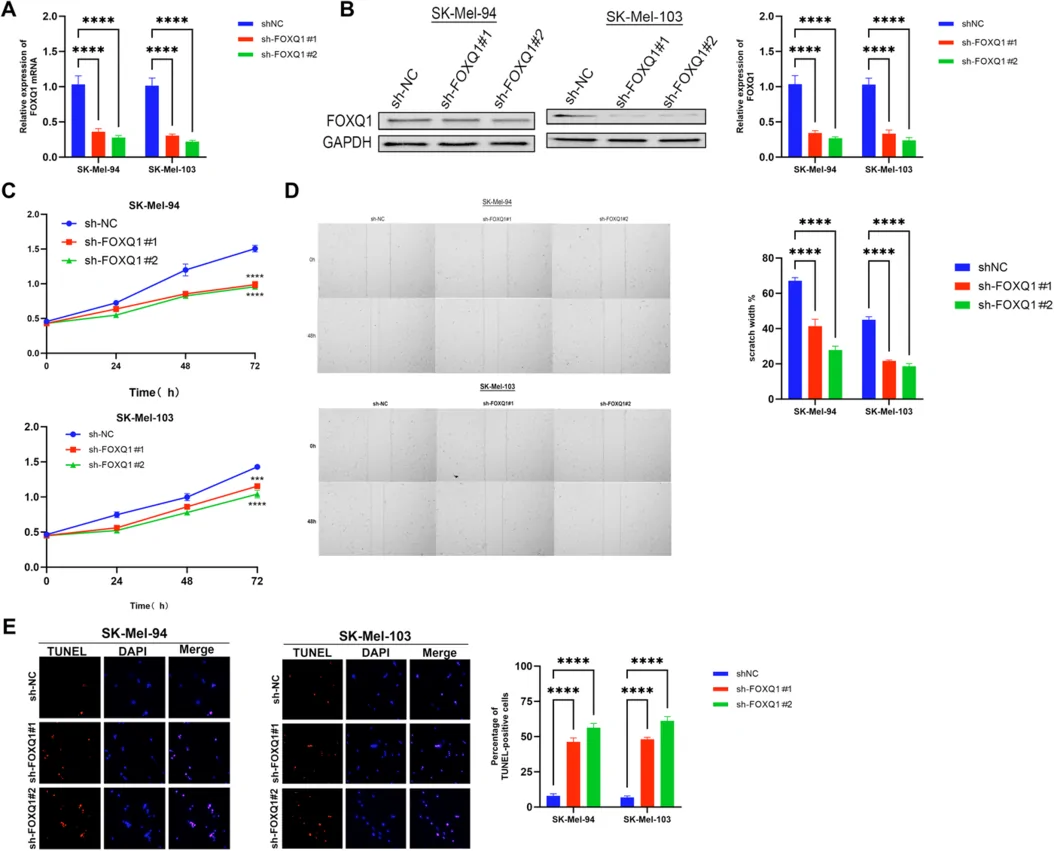
Silencing FOXQ1 suppresses malignant behaviors of melanoma cells in SK‐Mel‐94 and SK‐Mel‐103 cell lines. (A) FOXQ1 mRNA expression in SK‐Mel‐94 and SK‐Mel‐103 cells following FOXQ1 knockdown, as determined by quantitative real‐time PCR (qRT‐PCR). (B) FOXQ1 protein expression in SK‐Mel‐94 and SK‐Mel‐103 cells following FOXQ1 knockdown, as assessed by Western blot analysis. (C) Cell viability of SK‐Mel‐94 and SK‐Mel‐103 cells after FOXQ1 silencing measured by the Cell Counting Kit‐8 (CCK‐8) assay. (D) Wound healing assay showing changes in migratory capacity of SK‐Mel‐94 and SK‐Mel‐103 cells after FOXQ1 knockdown. (E) TUNEL staining assay showing apoptotic cells in SK‐Mel‐94 and SK‐Mel‐103 cell lines following FOXQ1 silencing (***p < 0.001; ****p < 0.0001, *n* = 3.).

### 3.7 FOXQ1 Exerts Transcriptional Repression on SSBP2 in Malignant Melanoma

Previous findings demonstrated that FOXQ1 is closely associated with multiple malignant behaviors of melanoma; however, the downstream regulatory mechanisms underlying its oncogenic effects remained unclear. To investigate the molecular pathways regulated by FOXQ1 in malignant melanoma (MM), downstream target genes of FOXQ1 were first predicted using the hTFtarget database. Differential expression analysis was subsequently performed using the TCGA‐SKCM cohort, resulting in the identification of six overlapping candidate target genes. Among these candidates, SSBP2 was identified as a prognosis‐related gene exhibiting significant differential expression. Kaplan–Meier survival analysis revealed that low SSBP2 expression was significantly associated with shortened OS in MM patients (log‐rank test, *p* = 0.0043; Figure 7A). In addition, SSBP2 expression was markedly reduced in MM tumor tissues compared with nonmalignant melanocytic tissues (Figure 7B). Based on these observations, experimental validation was subsequently performed. qRT‐PCR and Western blot analyses confirmed that SSBP2 expression was significantly downregulated in clinical MM tissues. Consistently, SSBP2 expression was also significantly lower in three MM cell lines (SK‐Mel‐28, SK‐Mel‐94, and SK‐Mel‐103) compared with normal human melanocytes, at both the mRNA and protein levels (Figure 7C,D). These results suggested a potential negative regulatory relationship between FOXQ1 and SSBP2, although the precise upstream–downstream regulatory mechanism required further clarification. To further elucidate the regulatory interaction between FOXQ1 and SSBP2 during MM progression, the SK‐Mel‐103 cell line, which exhibited the lowest SSBP2 expression, was selected for mechanistic studies. ChIP assays, qRT‐PCR analysis, and dual‐luciferase reporter assays were performed to validate the regulatory relationship between these two genes. ChIP analysis demonstrated that the relative enrichment of the SSBP2 promoter region in the FOXQ1 antibody group was significantly higher than that in the IgG negative control group, indicating that FOXQ1 specifically binds to the promoter region of the SSBP2 gene (Figure 7E). Furthermore, qRT‐PCR analysis showed that silencing FOXQ1 led to a significant increase in SSBP2 mRNA expression compared with the control group, further supporting a negative regulatory relationship between FOXQ1 and SSBP2 and suggesting that FOXQ1 functions as a transcriptional repressor of SSBP2 (Figure 7F). Consistently, dual‐luciferase reporter assays revealed that FOXQ1 knockdown significantly increased relative luciferase activity compared with the negative control, confirming that FOXQ1 directly binds to the SSBP2 promoter and that silencing FOXQ1 relieves its transcriptional repression of SSBP2, thereby influencing the malignant progression of MM (Figure 7G).

**Figure 7:**
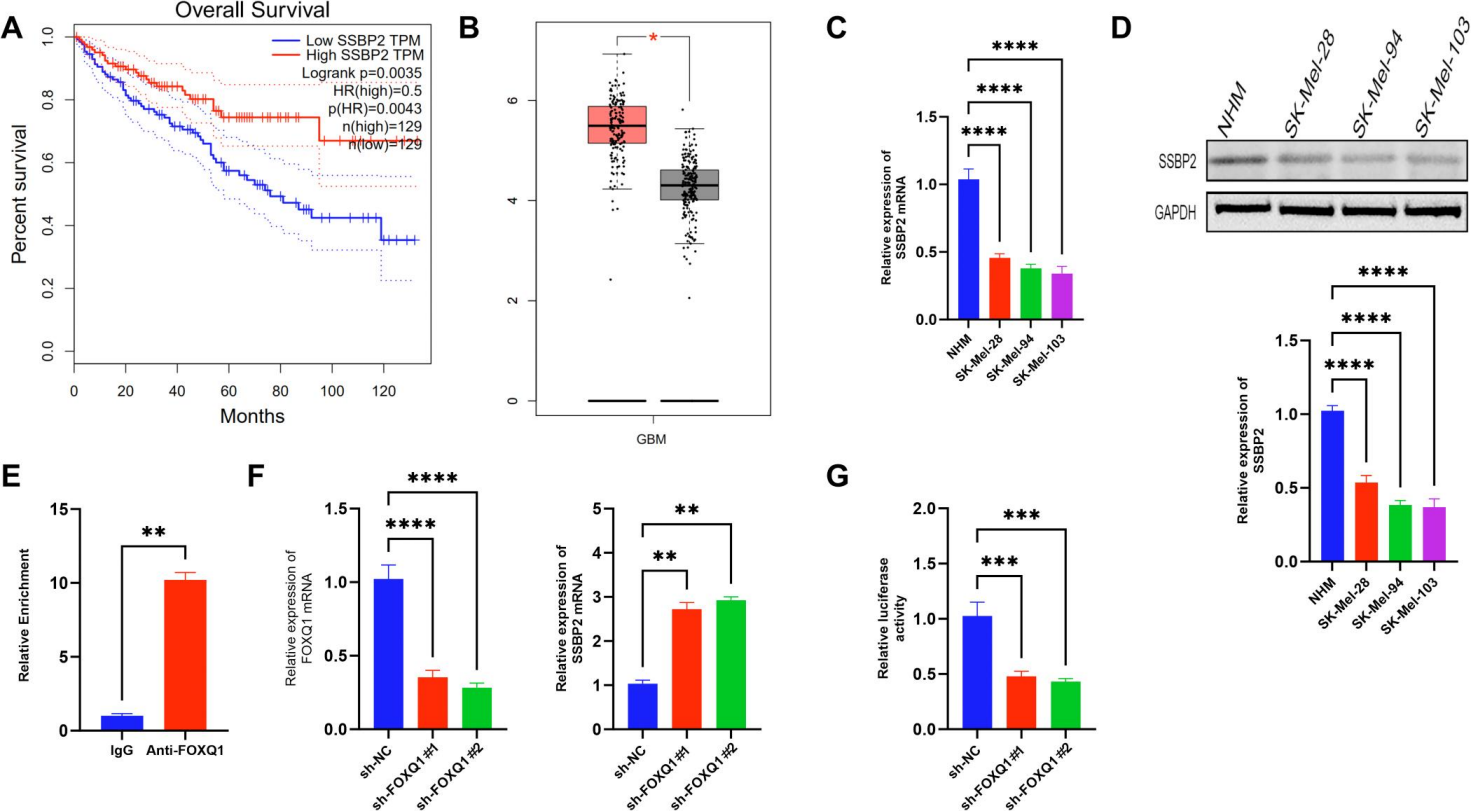
Silencing FOXQ1 transcriptionally activates SSBP2 expression in malignant melanoma cells. (A) Kaplan–Meier survival curves stratified by high and low SSBP2 expression levels. (B) Differential expression of SSBP2 in melanoma patients and normal controls. (C) SSBP2 mRNA expression levels in melanoma cell lines and normal human melanocytes measured by quantitative real‐time PCR (qRT‐PCR). (D) SSBP2 protein expression levels in melanoma cell lines and normal human melanocytes assessed by Western blot analysis. (E) Chromatin immunoprecipitation (ChIP) assay showing relative enrichment of the SSBP2 promoter region in SK‐Mel‐103 cells. (F) qRT‐PCR analysis of FOXQ1 and SSBP2 mRNA expression levels following FOXQ1 knockdown. (G) Dual‐luciferase reporter assay evaluating the interaction between FOXQ1 and the SSBP2 promoter following FOXQ1 silencing (**p < 0.01; ***p < 0.001; ****p < 0.0001, *n* = 3).

### 3.8 Downregulation of SSBP2 Enhances Malignant Behaviors of Melanoma Cells

To determine whether FOXQ1 contributes to malignant melanoma (MM) progression through the regulation of SSBP2, SSBP2 expression was silenced in the SK‐Mel‐103 cell line using lentiviral transduction. qRT‐PCR and Western blot analyses confirmed that both SSBP2 mRNA and protein levels were significantly reduced compared with the negative control group, indicating successful knockdown of SSBP2 (Figure 8A,B). Following SSBP2 silencing, the malignant behaviors of melanoma cells were markedly enhanced. The CCK‐8 assay demonstrated a significant increase in cell viability after SSBP2 knockdown (Figure 8C). Wound healing assays revealed that SSBP2 silencing significantly promoted the migratory capacity of SK‐Mel‐103 cells, while TUNEL staining showed a pronounced reduction in apoptotic activity in SSBP2‐deficient cells (Figure 8D,E). Collectively, these results indicate that downregulation of SSBP2 promotes malignant phenotypes in melanoma cells, supporting the notion that SSBP2 functions as a tumor suppressor in MM and mediates, at least in part, the oncogenic effects of FOXQ1.

**Figure 8:**
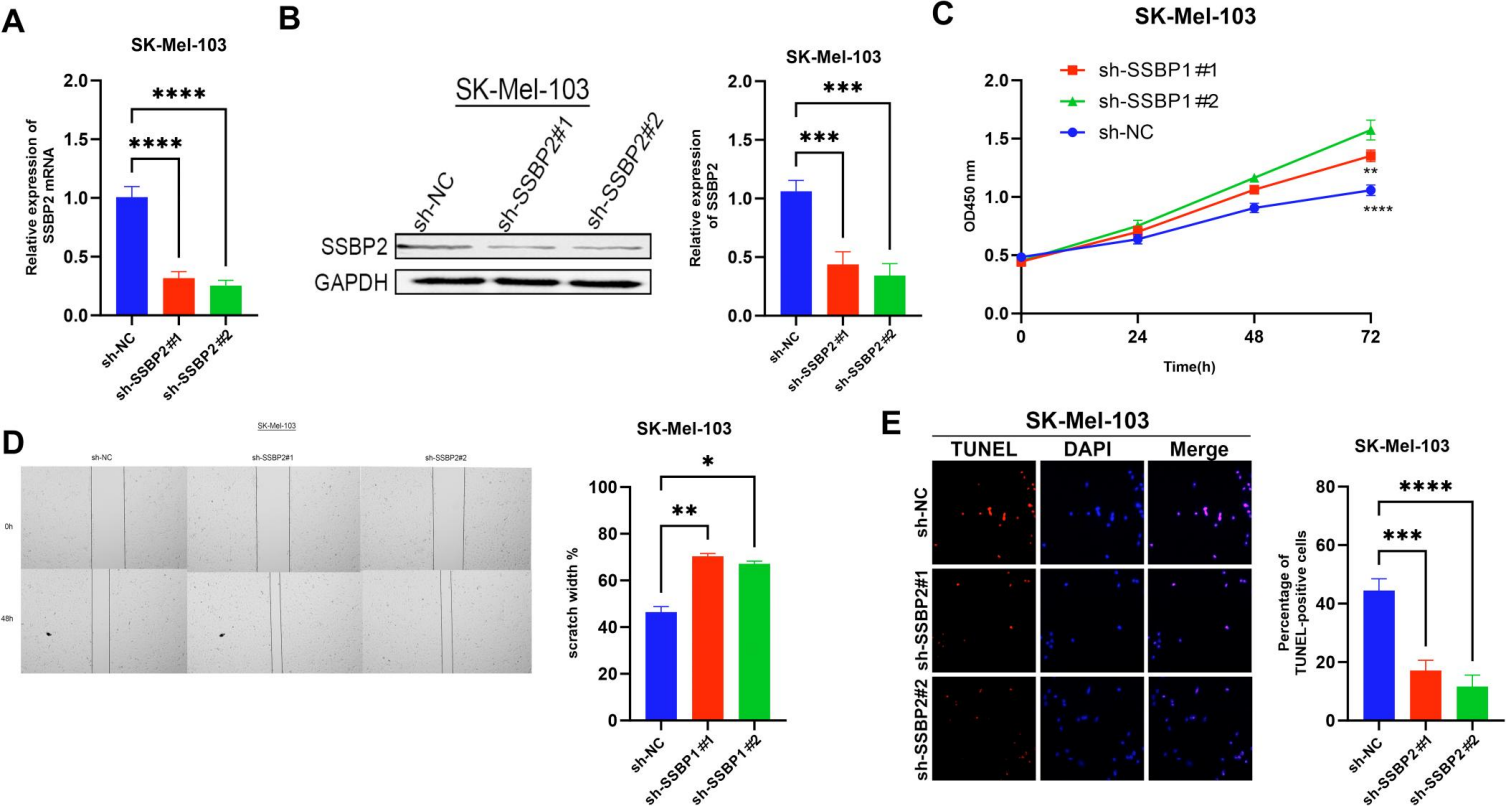
Downregulation of SSBP2 enhances the malignant behaviors of melanoma cells. (A) SSBP2 mRNA expression in SK‐Mel‐103 melanoma cells following SSBP2 knockdown, as determined by quantitative real‐time PCR (qRT‐PCR). (B) SSBP2 protein expression in SK‐Mel‐103 melanoma cells following SSBP2 knockdown, as assessed by Western blot analysis. (C) Cell viability of SK‐Mel‐103 cells after SSBP2 silencing measured by the Cell Counting Kit‐8 (CCK‐8) assay. (D) Wound healing assay showing changes in migratory capacity of SK‐Mel‐103 cells following SSBP2 knockdown. (E) TUNEL staining assay showing apoptotic cells in SK‐Mel‐103 cells after SSBP2 silencing (*p < 0.05; **p < 0.01; ***p < 0.001; ****p < 0.0001).

### 3.9 Rescue Experiments Confirm That FOXQ1‐Mediated Transcriptional Repression of SSBP2 Drives Malignant Progression of Melanoma

To further validate the coordinated regulatory relationship between FOXQ1 and SSBP2 during malignant melanoma (MM) progression, combined rescue experiments were performed. qRT‐PCR and Western blot analyses demonstrated that, in SK‐Mel‐103 cells, additional silencing of SSBP2 on the background of FOXQ1 knockdown significantly reversed the upregulation of SSBP2 at both the mRNA and protein levels. These findings further confirm that SSBP2 is a downstream target gene of FOXQ1 (Figure 9A,B). To determine the functional significance of this regulatory axis in melanoma malignancy, a series of phenotypic assays was conducted. Colony formation assays revealed that SSBP2 silencing markedly restored the proliferative capacity of melanoma cells that had been suppressed by FOXQ1 knockdown, with the observed changes reaching statistical significance (Figure 9C). Transwell invasion assays showed that additional knockdown of SSBP2 significantly rescued the invasive ability of melanoma cells under FOXQ1‐silenced conditions (Figure 9D). Furthermore, TUNEL assays demonstrated that SSBP2 silencing attenuated the pro‐apoptotic effects induced by FOXQ1 knockdown, resulting in a significant reduction in tumor cell apoptosis (Figure 9E). To investigate whether this regulatory mechanism also operates in vivo, a mouse xenograft tumor model was established. The results showed that additional SSBP2 knockdown on the background of FOXQ1 silencing significantly accelerated tumor growth, as evidenced by increased tumor volume and tumor weight (Figure 9F). To confirm that the observed in vivo phenotypic changes were mediated through FOXQ1‐dependent regulation of SSBP2, immunohistochemical analysis was performed to assess SSBP2 expression in xenograft tumors. The results demonstrated that SSBP2 expression was markedly reduced in tumors with combined FOXQ1 and SSBP2 knockdown compared with FOXQ1 knockdown alone (Figure 9G).

**Figure 9:**
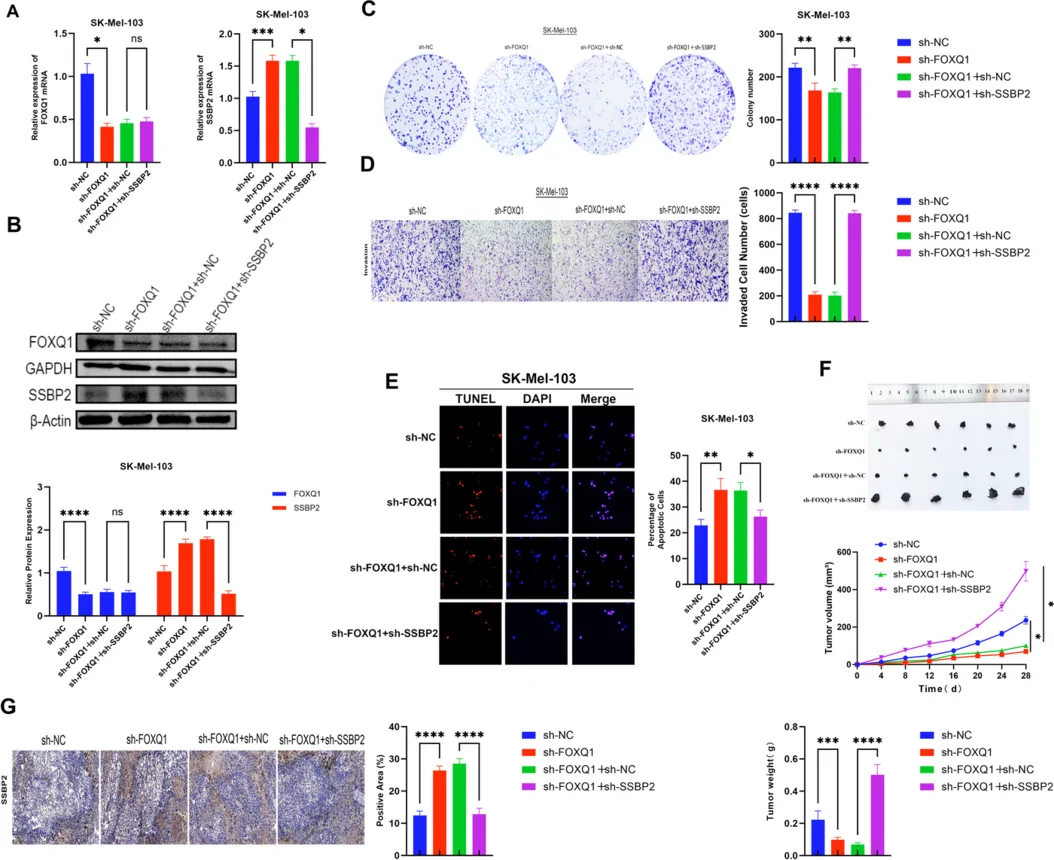
Rescue experiments demonstrate that FOXQ1‐mediated transcriptional repression of SSBP2 drives malignant progression of melanoma. (A) qRT‐PCR analysis of FOXQ1 and SSBP2 mRNA expression levels in SK‐Mel‐103 cells under different treatment conditions (*n* = 3). (B) Western blot analysis of FOXQ1 and SSBP2 protein expression levels in SK‐Mel‐103 cells (*n* = 3). (C) Colony formation assay evaluating clonogenic capacity of SK‐Mel‐103 cells (*n* = 3). (D) Transwell invasion assay assessing the invasive ability of SK‐Mel‐103 cells (*n* = 3). (E) TUNEL staining assay evaluating apoptotic activity of SK‐Mel‐103 cells (*n* = 3). (F) Tumor growth curves, tumor volume, and tumor weight changes in mouse xenograft models (*n* = 6). (G) Immunohistochemical staining showing SSBP2 protein expression in xenograft tumor tissues (ns, no significance; *p < 0.05; **p < 0.01; ***p < 0.001; ****p < 0.0001).

Collectively, these in vitro and in vivo findings demonstrate that FOXQ1 promotes malignant progression of melanoma through transcriptional repression of SSBP2, establishing the FOXQ1/SSBP2 regulatory axis as a critical driver of melanoma aggressiveness.

## 4 Discussion

This study, through a combination of systematic bioinformatics analyses and comprehensive in vitro and in vivo experimental validation, is the first to elucidate the critical role of the FOXQ1/SSBP2 regulatory axis in the malignant progression of melanoma. Bioinformatics analyses revealed that FOXQ1 consistently exhibited HRs > 1 across multiple melanoma data sets, indicating a strong association between FOXQ1 expression and poor clinical prognosis. Based on these findings, FOXQ1 was selected for functional silencing experiments.

FOXQ1 is a member of the Forkhead box (FOX) family of transcription factors and contains a conserved forkhead DNA‐binding domain that enables it to bind to target gene promoter regions and regulate transcription. Accumulating evidence has demonstrated that FOXQ1 exerts diverse biological functions across different tumor types [27]. In epithelial‐derived malignancies, including breast cancer, colorectal cancer, and intrahepatic cholangiocarcinoma, FOXQ1 is predominantly characterized as an oncogene. In these contexts, FOXQ1 promotes tumor invasion and metastasis by inducing EMT, modulating the Wnt/β‐catenin signaling pathway, and regulating cancer stemness programs [28, 29, 30]. However, emerging studies have suggested that FOXQ1 may exhibit a tissue‐ or lineage‐dependent “functional switch” in certain tumor types. For instance, in melanoma, alterations in FOXQ1 expression and function appear to differ from those observed in epithelial cancers. Its regulatory role may be constrained by the relative nuclear abundance of β‐catenin and transcriptional corepressors such as TLE, resulting in distinct transcriptional outcomes [31]. In the present study, we demonstrate that FOXQ1 is highly expressed in melanoma and functions as a pro‐oncogenic factor, consistent with its role in the majority of epithelial tumors. These findings indicate that FOXQ1 retains the capacity to positively regulate malignant phenotypes in melanoma cells. Aberrant upregulation of FOXQ1 in melanoma may be associated with chromosomal structural alterations, dysregulated transcriptional control, or melanoma‐specific epigenetic remodeling. Nevertheless, the precise upstream mechanisms responsible for FOXQ1 overexpression in melanoma remain to be elucidated and warrant further investigation.

SSBP2 was initially identified as a tumor suppressor gene in the hematopoietic system, where it plays a critical role in maintaining genomic stability and hematopoietic stem cell homeostasis. Its N‐terminal domain is capable of interacting with LDB1, thereby regulating the assembly and function of transcriptional complexes [17]. Accumulating evidence indicates that SSBP2 exhibits context‐dependent, bidirectional functions across different tumor types. In malignancies such as leukemia, colorectal cancer, and head and neck squamous cell carcinoma, SSBP2 is frequently downregulated and functions as a tumor suppressor [32]. In contrast, in glioblastoma and hepatocellular carcinoma, SSBP2 has been reported to promote tumor initiation and progression [24, 33]. These opposing roles are likely attributable to differences in the composition and regulatory dynamics of transcriptional complexes across distinct cellular lineages and tumor contexts. In the present study, SSBP2 was found to be significantly downregulated in melanoma tissues and cell lines. Functional assays further demonstrated that silencing SSBP2 markedly enhanced melanoma cell viability and migratory capacity while suppressing apoptotic activity. Collectively, these findings indicate that SSBP2 functions as a tumor suppressor in melanoma. Downregulation of SSBP2 significantly promotes melanoma cell proliferation and migration and inhibits apoptosis, thereby facilitating malignant progression. These observations are consistent with the tumor‐suppressive role of SSBP2 reported in myeloid leukemia and colorectal cancer, further supporting the notion that SSBP2 exerts a broadly inhibitory effect on tumor progression in a context‐dependent manner.

This study is the first to demonstrate that FOXQ1 directly binds to the promoter region of SSBP2 and suppresses its transcriptional activity. Combined rescue experiments further revealed that additional silencing of SSBP2 on the background of FOXQ1 knockdown reverses the tumor‐suppressive phenotypes induced by FOXQ1 depletion, indicating that FOXQ1 exerts its oncogenic effects through negative regulation of SSBP2. From a transcriptional regulatory perspective, FOXQ1 may recognize and occupy critical sequences within the SSBP2 promoter via its conserved forkhead DNA‐binding motif (FHA motif), thereby obstructing the recruitment of the RNA polymerase II complex or facilitating the assembly of transcriptional repressor complexes, such as HDAC‐ or TLE‐containing corepressor complexes, to inhibit SSBP2 transcription [31]. This regulatory process may be influenced by the melanoma‐specific transcriptional cofactor landscape, a hypothesis that warrants further mechanistic investigation.

Consistent results from in vitro assays (including CCK‐8, wound healing, Transwell invasion, and TUNEL assays) and in vivo experiments (mouse xenograft tumor models) collectively demonstrate that the FOXQ1/SSBP2 axis plays a decisive role in regulating the malignant behaviors of melanoma cells. Upregulation of FOXQ1 suppresses SSBP2 expression, thereby relieving constraints on tumor cell proliferation and migration and ultimately promoting melanoma growth and metastasis. This mechanism reveals a previously unrecognized layer of transcriptional regulation in melanoma biology. Unlike classical melanoma driver mutations such as BRAF, NRAS, and c‐KIT [34, 35], the FOXQ1/SSBP2 axis represents an epigenetic and transcriptional regulatory mechanism rather than a direct genetic alteration. This axis may contribute to tumor aggressiveness, therapeutic resistance, and immune evasion, thereby providing a novel conceptual framework and potential molecular target for melanoma treatment strategies.

In summary, by integrating systematic bioinformatics analyses with comprehensive experimental validation, this study demonstrates that FOXQ1 is a key transcription factor driving melanoma progression, and that SSBP2 is a direct downstream target transcriptionally repressed by FOXQ1. Both in vitro and in vivo experiments consistently confirmed that FOXQ1 promotes the malignant behaviors of melanoma cells through suppression of SSBP2 expression. These findings establish the FOXQ1/SSBP2 regulatory axis as a critical molecular mechanism underlying melanoma aggressiveness. Investigation of the FOXQ1/SSBP2 axis may therefore represent a promising therapeutic strategy for improving disease progression and unfavorable prognosis in malignant melanoma. However, several limitations of the present study should be acknowledged. First, further validation at the clinical level is required to substantiate the translational relevance of these findings. Second, although the transcriptional regulatory relationship between FOXQ1 and SSBP2 was established, the downstream signaling pathways and molecular networks governed by the FOXQ1/SSBP2 axis were not fully elucidated. Future studies should focus on dissecting the specific signaling pathways and pathological phenotypes modulated by this regulatory axis in melanoma progression. In addition, expanded clinical and translational studies are warranted to further explore the diagnostic and therapeutic potential of targeting the FOXQ1/SSBP2 axis in melanoma.

## Author Contributions

Conceptualization: Xiaoyu Zhang and Yu Hou. Data curation: Xiaoyu Zhang, Yu Hou, Jiahui Liu, and Yuehua Han. Formal analysis: Xiaoyu Zhang, Yu Hou, and Yuehua Han. Funding acquisition: Jiangang Feng. Investigation: Xiaoyu Zhang, Yu Hou, and Yuehua Han. Methodology: Xiaoyu Zhang, Yu Hou, and Jiahui Liu. Project administration: Xiaoyu Zhang and Yu Hou. Resources: Jiangang Feng. Software: Xiaoyu Zhang, Yu Hou, and Jiahui Liu. Supervision: Jiangang Feng. Validation: Xiaoyu Zhang, Yu Hou, and Jiahui Liu. Visualization: Xiaoyu Zhang and Yu Hou. Writing – original draft: Xiaoyu Zhang, Yu Hou, Jiahui Liu, and Yuehua Han. Writing – review and editing: Jiangang Feng. All authors have read and agreed to the published version of the manuscript.

## Ethics Statement

This study was approved by the Ethics Committee of The Fourth Hospital of Hebei Medical University (Approval Number: 20221731).

## Consent

Written informed consent was obtained from all patients prior to surgery. Patient consent for publication was not required as this study used anonymized data and contained no identifiable personal information.

## Conflicts of Interest

The authors declare no conflicts of interest.

## Supporting information


Supporting File

## Data Availability

The data that support the findings of this study are available within the article. The original data can be obtained by sending an email to the corresponding author.
