# Supplementary material for: The FOXQ1/SSBP2 Regulatory Axis Drives Malignant Progression of Melanoma
Source: Mol Carcinog. 2026 Aug 30;65(11):1459–77. doi: 10.1002/mc.70126 (PMC13656191; doi:10.1002/mc.70126)
Supplement: Supplementary file 1 [file MC-65-1459-s001.docx]

***The FOXQ1/SSBP2 regulatory axis drives malignant progression of melanoma***

Supplementary Table 1.Target sequences used for gene silencing

| Gene | Construct name | Target sequence (5′–3′) | Type |
| --- | --- | --- | --- |
| FOXQ1 | sh-FOXQ1#1 | Manufacturer-designed shRNA | shRNA |
|  | sh-FOXQ1#2 | Manufacturer-designed shRNA |  |
| SSBP2 | sh-SSBP2#1 | Manufacturer-designed shRNA | shRNA |
|  | sh-SSBP2#2 | Manufacturer-designed shRNA |  |
